# Increased Oxidative Damage in Carriers of the Germline *TP53* p.R337H Mutation

**DOI:** 10.1371/journal.pone.0047010

**Published:** 2012-10-09

**Authors:** Gabriel S. Macedo, Leonardo Lisbôa da Motta, Juliana Giacomazzi, Cristina B. O. Netto, Vanusa Manfredini, Camila S.Vanzin, Carmen Regla Vargas, Pierre Hainaut, Fábio Klamt, Patricia Ashton-Prolla

**Affiliations:** 1 Programa de Pós-Graduação em Genética e Biologia Molecular, Universidade Federal do Rio Grande do Sul (UFRGS), Porto Alegre, Brazil; 2 Laboratório de Medicina Genômica, Hospital de Clínicas de Porto Alegre (HCPA), Porto Alegre, Brazil; 3 Programa de Pós-Graduação em Ciências Biológicas: Bioquímica, UFRGS, Porto Alegre, Brazil; 4 Institutos Nacionais de Ciência e Tecnologia - Translacional em Medicina (MCT/CNPq INCT-TM), Porto Alegre, RS, Brazil; 5 Rede Gaúcha de Estresse Oxidativo e Sinalização Celular (FAPERGS), Porto Alegre, RS, Brazil; 6 Programa de Pós-Graduação em Ciências Médicas: Medicina, UFRGS, Porto Alegre, Brazil; 7 Serviço de Genética Médica, HCPA, Porto Alegre, Brazil; 8 Universidade Federal do Pampa (UNIPAMPA), Campus Uruguaiana-RS, Brazil; 9 International Prevention Research Institute, Lyon, France; Virginia Commonwealth University, United States of America

## Abstract

Germline mutations in *TP53* are the underlying defect of Li-Fraumeni Syndrome (LFS) and Li-Fraumeni-like (LFL) Syndrome, autosomal dominant disorders characterized by predisposition to multiple early onset cancers. In Brazil, a variant form of LFS/LFL is commonly detected because of the high prevalence of a founder mutation at codon 337 in *TP53* (p.R337H). The p53 protein exerts multiple roles in the regulation of oxidative metabolism and cellular anti-oxidant defense systems. Herein, we analyzed the redox parameters in blood samples from p.R337H mutation carriers (C, n = 17) and non-carriers (NC, n = 17). We identified a significant increase in erythrocyte GPx activity and in plasma carbonyl content,an indicator of protein oxidative damage, in mutation carriers compared to non-carriers (*P* = 0.048 and *P* = 0.035, respectively). Mutation carriers also showed a four-fold increase in plasma malondialdehyde levels, indicating increased lipid peroxidation (NC = 40.20±0.71, C = 160.5±0.88, *P*<0.0001). Finally, carriers showed increased total antioxidant status but a decrease in plasma ascorbic acid content. The observed imbalance could be associated with deregulated cell bioenergetics and/or with increased inflammatory stress, two effects that may result from loss of wild-type p53 function. These findings provide the first evidence that oxidative damage occurs in carriers of a germline *TP53* mutation, and these may have important implications regarding our understanding of the mechanisms responsible for germline *TP53* p.R337H mutation-associated carcinogenesis.

## Introduction

Li-Fraumeni Syndrome (LFS) and its variant, Li-Fraumeni-like Syndrome (LFL), are autosomal dominant disorders characterized by increased predisposition to multiple early-onset cancers [1]. The core tumors of the syndrome are soft tissue and bone sarcomas, brain tumors, breast cancer, and adrenocortical carcinoma (ACC). Other malignancies such as leukemia, melanomas, and lung, gastric, pancreatic, prostate, and colorectal cancers have also been described in several families [2]–[5]. The clinical definition of LFS/LFL is based on tumor patterns present in the proband and in first and second degree relatives. Currently, germline mutations in the *TP53* gene are the only known genetic defect underlying LFS/LFL. About 56% of the families that meet the strict “classic” criteria for LFS have germline *TP53* mutations. In LFL families, mutations are detected in about 36%, 16%, and 14% of probands that meet criteria of Chompret, Birch, and Eeles, respectively [6].

The p53 nuclear phosphoprotein acts primarily as a transcription factor. Its main role as a tumor suppressor is to operate in the cellular responses to a wide range of stress signals such as oncogenic activation and DNA damage [7]. Structurally, it consists of a homotetramer, and each of its monomers is composed of 393 amino acids. Well-defined domains can be identified in the protein, including an N-terminal transactivation domain, a proline-rich region, a central and highly conserved DNA-binding domain (DBD), a tetramerization domain, and unstructured basic domains at its C-terminus [8]. Activated p53 suppresses cellular transformation by regulating the expression of an array of different genes, encoding both proteins and microRNAs, and is involved in growth arrest, DNA repair, apoptosis, and senescence pathways [9]. Recently, p53 has been shown to regulate many aspects of energy metabolism as well as enzymes that are involved in cell responses to oxidative stress [10]. Genes such as mitochondrial superoxide dismutase 2 (*SOD2*) [11], glutathione peroxidase 1 (*GPX1*) [12], aldehyde dehydrogenase 4 family member A1 (*ALDH4A1*) [13], and some of the sestrin family genes (*HI95* and *PA26*) [14] encode products that act as antioxidants and are modulated by p53. In 2005, Sablina et al. presented convincing evidence supporting that the antioxidant functions of low or basal levels of p53 have a role in the control of genetic stability and prevention of cancer [15]. However, thus far, there has been no published evidence that carriers of germline *TP53* mutations harbor differences in their redox metabolism and anti-oxidant responses compared to non-carriers.

The most common *TP53* germline mutations that are associated with LFS and LFL are substitutions clustering in highly conserved regions of the DBD. Inheritance of such mutations is generally associated with severe forms of LFS/LFL. However, in rare instances, missense mutations occur outside of the DBD. Among these, is the p.R337H (c.1010 G>A, CGC to CAC at codon 337) mutation in exon 10, which was initially reported in Brazilian children diagnosed with ACC but no documented familial history of other cancers [16], [17]. Subsequently, p.R337H has been associated with a broader cancer spectrum, similar to that of LFS/LFL, including premenopausal breast cancer, early sarcoma, and other tumors that occur at an earlier age in carriers when compared to non-carriers [18], [19]. Strikingly, this mutation is present in 0.3% of the general population in Southern Brazil [20], likely due to a founder effect [21]. Unlike other common LFS mutants, which dramatically disrupt p53 transcriptional activity, p.R337H is thought to partially retain the ability to suppress cell growth under certain physiological conditions. Structural studies have shown that the mutation hampers p53 oligomerization in a pH-dependent manner [22]. Thus, at pH 7, p.R337H retains its capacity to form oligomers and exhibits a structure and activity that are similar to wild-type p53. At slightly elevated pH, however (pH 8.0), p.R337H loses its capacity to form oligomers and thus to bind to p53 DNA response elements despite retaining a structurally intact DBD. This biochemical property defines p.R337H as being a dysfunctional pH-dependent p53 mutant [23].

Given the relatively high population prevalence of this mutant allele in southern Brazil and its association with increased risk of developing several cancers, p.R337H may make an important contribution to the cancer burden in the region. Moreover, this particular context provides the possibility for assembling studies on apparently unrelated subjects who all carry the same mutation, thus limiting baseline variations that may result from widely different mutations. We therefore analyzed the levels of several markers of oxidative stress and of enzymes that are involved in oxidative stress responses in blood samples of p.R337H mutation carriers and non-carriers. In doing so, we sought baseline changes in the oxidative stress status that could be potentially associated with the presence of this mutation in the germline.

## Materials and Methods

### Patients and Controls

A total of 34 individuals were recruited in the setting of a familial cancer clinic. The study was approved by the Institutional Ethics Committee of Hospital de Clínicas de Porto Alegre under protocol number 11–0099. All of the participants and/or their legal representatives were recruited only after providing informed consent. Two groups of 17 subjects were assembled (Table 1 and Table S1). Group C (p.R337H carriers) comprised subjects who had previously been ascertained as being carriers of the p.R337H mutation in genomic (white blood cells) DNA by standard sequencing protocols (http://www-p53.iarc.fr/Download/TP53_DirectSequencing_IARC.pdf). Group NC (non-p.R337H carriers) comprised 17 subjects who were ascertained as being non-carriers of the p.R337H or of any other mutation within the TP53 coding sequence (exons 2–11). The two groups were structured to have similar compositions with respect to gender, age, and history of disease. Among the 17 Group C subjects, 6 had less than 18 years (pediatric carriers) and 11 were adults (adult carriers). Of the 6 pediatric carriers, one was identified as being homozygous for the mutation, having inherited the mutant allele of both parents. Five of the carriers including the homozygous patient, had developed cancer. Among the pediatric carriers, 3 developed ACC and 1 developed CPT during the first year of life; among adults, one carrier had been diagnosed with breast cancer at the age of 35 years. The 12 other Group C subjects has no personal histories of cancer. The Group NC subjects included 12 relatives of the p.R337H carriers that were included in Group C, all without personal histories of cancer, and 5 subjects who had histories of cancer, including one patient with ACC who had no detectable germline TP53 mutation determined by sequencing of the entire coding region. This patient was posteriorly diagnosed with Beckwith–Wiedemann syndrome. All patients and subjects in Groups C and NC were healthy and cancer-free at the time of sampling. For patients with a previous diagnosis of cancer, the mean interval between diagnosis and inclusion in this study was 78 months, and all the patients were in remission and/or asymptomatic for at least 36 months.

**Table 1:** Gender, mean age and number of individuals with cancer diagnosis among *TP53* p.R337H mutation carriers and non-carriers.

Mutation status	Gender (n)^a^	Mean age(range)^b^	Cancer-affectedindividuals n (%)	Cancer diagnoses (n)^c^
Carriers (n = 17)	M (7), F (10)	25 (3–52)	5 (29.4)	ACC (n = 3), CPT (n = 1), DBC (n = 1)
Non-carriers (n = 17)	M (9), F (8)	27 (2–50)	5 (29.4)	ACC (n = 1), ALL (n = 1), AML (n = 1), GCT (n = 1) and MT (n = 1)

a M = male and F = female.

b in years.

c ACC (adrenocortical carcinoma), CPT (choroid plexus carcinoma), DBC (ductal breast carcinoma), ALL (acute lymphocytic leukemia), AML (acute myeloid leukemia), GCT (germ cell tumor) and MT (mature teratoma).

### Sample Preparation

Peripheral blood samples were obtained from all individuals under sterile conditions: 2 vials containing EDTA and 1 vial free of anti-coagulants. Whole blood was centrifuged at 1,000 × *g* for 10 min at 4°C and separated into plasma, erythrocytes, leukocytes, and serum. For antioxidant enzyme activities, erythrocytes and leukocytes were lysed by 3 sequential freezing and thawing cycles, followed by centrifugation at 1,300 × *g* for 15 min. The samples were kept frozen at −80°C until analysis. Protein concentrations were determined by standard methods [24]. Enzyme activity results were expressed as enzyme units/mg protein.

### Antioxidant Enzyme Assays

Superoxide dismutase (SOD; E.C. 1.15.1.1) activity in erythrocytes, leukocytes, plasma, and serum was measured by inhibiting superoxide-dependent epinephrine auto-oxidation at 480 nm [25]. One SOD unit was defined as the amount of sample that inhibited 50% of epinephrine auto-oxidation.

Catalase (CAT; E.C. 1.11.1.6) activity in erythrocytes was assayed by H_2_O_2_ consumption at 240 nm [26]. One unit of the enzyme was defined as 1 µmol H_2_O_2_ consumed/min.

Glutathione peroxidase (GPx; E.C. 1.11.1.9) activity in erythrocytes and leukocytes was measured by NADPH oxidation at 340 nm in a system containing glutathione, glutathione reductase, and *tert*-butyl hydroperoxide as substrate [27]. One GPx unit was defined as NADPH (nmol) oxidized/min.

### Total Anti-oxidant Status

Total anti-oxidant status (TAS) was determined in plasma using the TAS® Kit (Randox, Antrim, United Kingdom). ABTS® (2.2 -azino-di-[3-ethylbenzthiazoline sulphonate]) was incubated with peroxidase and H_2_O_2_ to produce the radical **ABTS®^+^**, which resulted in a relatively stable blue-green color measurable at 600 nm. Anti-oxidants suppress the production of this color to a degree that is proportional to their concentration [28]. The results were expressed in mmol/L plasma.

### Malondialdehyde Levels and Ascorbic Acid Content

Malondialdehyde (MDA) was measured simultaneously with ascorbic acid in plasma by HPLC [29]. One hundred milliliters of 0.1 M perchloric acid and 1 mL of distilled water were added to a 100-µL aliquot of human plasma. Perchloric acid was necessary to precipitate proteins and release the MDA bound to the amino groups of proteins and other amino compounds. The samples were then centrifuged at 1,500 × *g* for 5 min and used for HPLC analysis. The mobile phase was 82.5∶17.5 (v/v) 30 mM monobasic potassium phosphate (pH 3.6)-methanol, and the column used was Supelcosil C18 (5 mm particle size) 15 cm 4.6 mm. The flow rate was 1.2 mL/min, and the chromatograms were monitored at 250 nm. Results were expressed in mmol.

### Protein Carbonyl Content

Duplicate aliquots (500 µL of plasma) of each sample were supplemented with 500 µL of 10 Mm 2.4-dinitrophenylhydrazine or 1.0 mL of 2 M HCl (blank tube) and 50% trichloroacetic acid. After 30 min, 250 µL of 50% trichloroacetic acid was added. The samples were centrifuged at 8,000 × *g* for 30 min to obtain the protein pellets, which were immediately washed with ethanol-ethyl acetate 1∶1 (v/v). The final protein pellets were diluted in 500 µL of 8 M urea buffer and incubated at 50°C for 90 min. Quantification was performed using a spectrophotometer at 370 nm [30]. Carbonyl (CO) content was calculated using the millimolar absorption coefficient of hydrazone (ε_370 nm_ = 21,000000 M^−1 ^cm^−1^), and the results were expressed in nmol CO/mg protein.

### Statistical Analyses

All of the analyses were performed using GraphPad Software Inc., San Diego, CA, USA, version 5.0. The data were expressed as mean ± standard error of mean of at least 2 independent experiments. The normal distribution was tested using the D’Agostino-Pearson omnibus test. Student’s *t*-test was used to analyze differences between groups. A *P* value<0.05 was considered statistically significant. Values that deviated by 2 standard deviations from the mean were excluded from analysis.

## Results

There were no statistically significant differences between the adults and pediatric subjects for any of the parameters that we evaluated (data not shown).

Erythrocyte GPx activity was significantly higher in Group C (mutation carriers) than in NC (non- carriers) (C = 9.23±0.81, NC = 6.99±0.58, *P* = 0.048). All the other enzyme activities did not differ between groups (Table 2).

**Table 2:** Antioxidant enzyme activities in blood from *TP53* p.R337H mutation carriers and non-carriers.

	Non-carriers	Carriers	*P* value
**Erythrocytes**			
SOD	7.15±0.52 (13)	8.16±0.85 (13)	0.323
CAT	24.70±1.42 (13)	24.14±0.83 (16)	0.728
GPx	6.99±0.58 (12)	9.23±0.81 (17)	0.048
**Leukocytes**			
SOD	40.49±0.49 (11)	40.02±0.63 (16)	0.593
GPx	6.29±0.65 (13)	8.12±0.79 (16)	0.093
**Plasma**			
SOD	32.09±0.69 (13)	31.67±0.50 (17)	0.616
**Serum**			
SOD	8.35±0.43 (9)	7.35±0.50 (11)	0.156

Data are presented as mean ± S.E.M. All results are expressed as enzyme units (U)/mg protein. Number of individuals considered after exclusion of measurements ±2 S.D from the mean are given in parenthesis.

**Figure 1:**
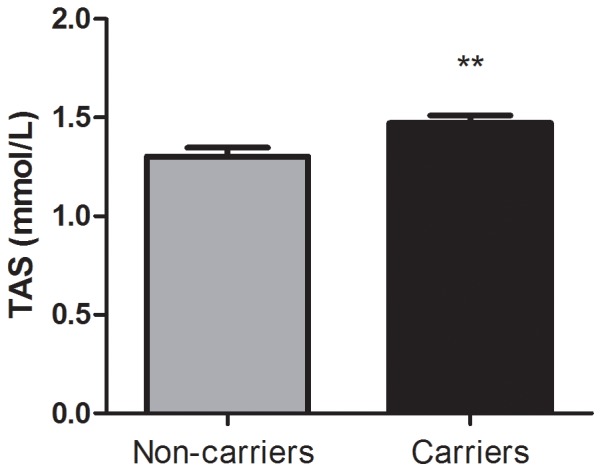
Total antioxidant status in plasma. Total antioxidant status (TAS) in plasma of *TP53* p.R337H carriers (n = 16) and non-carriers (n = 16). Data represent mean ± S.E.M (**) *P*<0.01 (Student *t* Test for unpaired samples).

**Figure 2:**
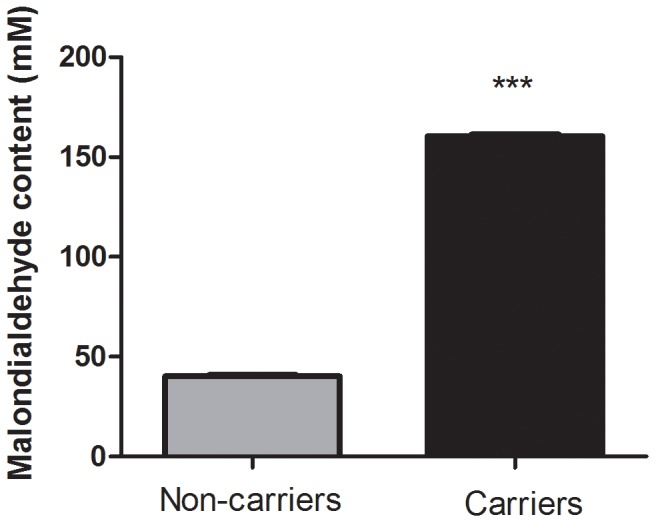
Malondialdehyde (MDA) levels in plasma. Malondialdehyde (MDA) levels in plasma from *TP53* p.R337H mutation carriers (n = 16) and non-carriers (n = 16). Data represent the mean ± S.E.M, (***) *P*<0.0001 (Student *t* Test for unpaired samples).

**Figure 3:**
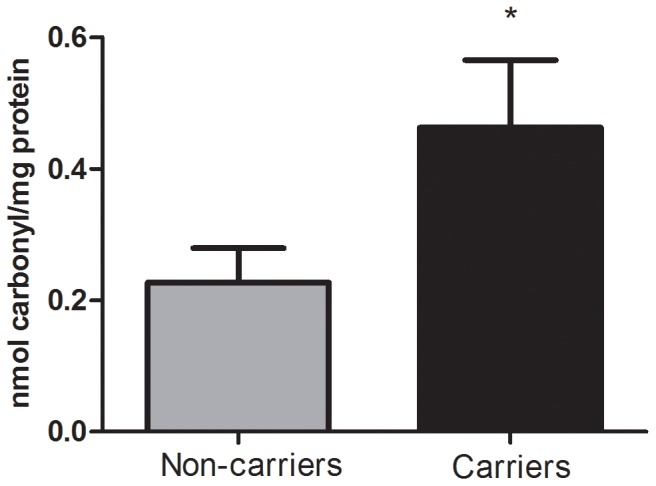
Carbonyl content in plasma. Carbonyl content (mean ± S.E.M) in plasma from p.R337H mutation carriers (n = 11) and non-carriers (n = 14). (*) *P*<0.05 (Mann Whitney test).

**Figure 4:**
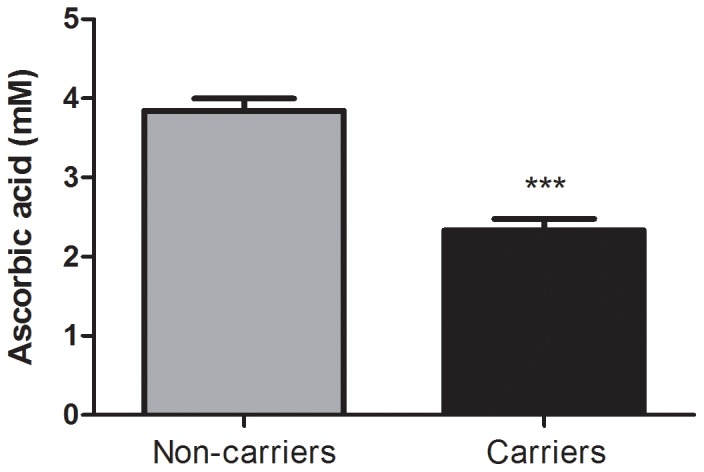
Ascorbic acid (vitamin C) content in plasma. Ascorbic acid content in plasma from *TP53* p.R337H mutation carriers (n = 16) and non-carriers (n = 16). Data represent the mean ± S.E.M, (***) *P*<0.0001 (Student *t* Test for unpaired samples).

The measure of total anti-oxidant status (TAS), which primarily represents non-enzymatic anti-oxidant levels, was significantly higher in carriers when compared to non-carriers (C = 1.47±0.04, NC = 1.30±0.04, *P* = 0.007; Figure 1). In addition, we observed an association between plasma MDA content and mutation status. Carriers had significantly higher MDA levels than non-carriers (C = 160.5±0.88, NC = 40.20±0.71, *P*<0.0001; Figure 2). Furthermore, the plasma CO content was increased in the carriers (C = 0.46±0.10, NC = 0.23±0.05, *P* = 0.035; Figure 3). Finally, ascorbic acid (vitamin C) levels were significantly lower in carriers when compared to non-carriers (C = 2.33±0.15, NC = 3.84±0.15, *P*<0.0001; Figure 4). When considering the personal histories of cancer in Groups C and NC, there was no significant difference between cancer affected and unaffected individuals in terms of ascorbic acid content, TAS, MDA levels, and CO content (Table 3). In the p.R337H homozygous patient included in Group C, the enzymatic and non-enzymatic anti-oxidant markers and oxidative damage indicators were comparable to those of the p.R337H/WT heterozygotes (Table S2).

**Table 3:** Ascorbic acid content, total antioxidant status, carbonyl content and malondialdehyde levels in *TP53* p.R337H mutation carriers and non-carriers with positive (PPH) and negative (NPH) personal histories of cancer.

	Non-carriers		*P*	Carriers		*P*
	NPH (n)	PPH (n)		NPH (n)	PH (n)	
Ascorbic acid content (mM)	3.90±0.21(11)	3.61±0.16(5)	0.370	2.39±0.19(11)	2.21±0.22(5)	0.583
TAS (mmol/L)	1.34±0.05(11)	1.22±0.08(5)	0.232	1.47±0.04(12)	1.47±0.10(4)	0.936
Carbonyl content(nmolcarbonyl/mg of protein)	0.20±0.06(10)	0.30±0.09(4)	0.419	0.52±0.13(8)	0.32±0.14(3)	0.408
Malondialdehyde levels(mmol/L)	40.09±0.93(11)	40.44±1.44(5)	0.827	159.6±1.04(11)	161.7±1.08(5)	0.316

Data are presented as mean ± S.E.M.

## Discussion

In recent years, a number of studies have shown that p53 operates as a regulator of cell oxidative metabolism and energy production [31]–[33]. On the other hand, p53 also regulates enzymes that are involved in the control of intracellular reactive oxygen species (ROS) and reactive nitrogen species (RNS), which are the main chemical mediators of oxidative stress [11]–[13], [34]. At the systemic level, oxidative stress refers to the imbalance between pro-oxidant and antioxidant states in tissues. ROS and RNS, which are primarily generated in the mitochondria, accumulate in cells, and they damage proteins, lipids, and DNA. They thereby trigger complex cellular responses that involve p53 as a mediator of cell growth, apoptosis, DNA repair, and cell senescence. Oxidative stress is the underlying biochemical condition in a number of chronic diseases including metabolic, cardiovascular, inflammatory, and neurodegenerative disorders, as well as cancer [35].

In this study, we measured several markers of oxidative stress and levels of the redox enzymes CAT, SOD, and GPx in the blood of clinically healthy subjects who all carry the same *TP53* germline mutation, and compared them to a group of non-carriers. Our study design took advantage of a unique situation that has arisen in southeastern Brazil, where many apparently unrelated subjects have inherited the same germline mutation due to a widespread founder effect [21]. This mutation occurs at codon 337 (G>A, CGC to CAC) and induces the substitution of an arginine for histidine in the tetramerization domain of p53 (p.R337H, c.1010). Since *TP53* mutations vary in their position in the coding sequence and their impact on p53 protein structure and function, this design had the advantage of reducing the baseline heterogeneity that could have occurred with the comparison of samples from subjects with different inherited mutations.

We have provided evidence suggesting that subjects who carry *TP53* p.R337H in their germline have higher levels of several blood indicators of oxidative stress than do subjects (matched by age and clinical history) who do not carry a germline *TP53* mutation. An increase in oxidative damage was detected in all p.R337H carriers irrespective of their previous cancer history, while it was not observed in subjects in the reference group. This observation suggests that altered levels of molecular damage caused by oxidative stress in blood products of *TP53* p.R337H carriers are related to the carcinogenic process and are not a consequence of malignancy. Whether the same biochemical phenotype occurs with germline *TP53* mutations other than p.R337H and whether it is part of the mechanisms that lead to increased cancer predisposition in this high-risk group remains to be determined.

Our results demonstrate an increased CO content and a four-fold increase in MDA, which are markers of protein oxidative damage and lipid peroxidation, respectively, in the plasma of *TP53* p.R337H carriers versus non-carriers. Lipid peroxidation may cause significant changes in the permeability, fluidity, integrity, and functional abnormalities of biomembranes, resulting in altered cell signaling and in the release of other potentially toxic cellular products [35]. On the other hand, the oxidation of protein side chains, especially those of Pro, Arg, Lys, and Thr results in the production of CO groups and altered protein structure and function. [36]. Although there is compelling evidence from cohort studies that increased levels in a wide range of markers of oxidative stress and inflammation are associated with an increased risk of cancer [37], there is only few published data on baseline oxidative stress parameters for individuals with cancer-predisposing germline mutations in tumor suppressor genes. To the best of our knowledge, only one study assessed the serum oxidative stress parameters of cancer-unaffected carriers of germline *BRCA1* mutations and found that the mutation status did not influence lipid and protein peroxidation content when compared to controls [38].

In addition to the evidence regarding oxidative damage, we also found higher erythrocyte GPx activity in *TP53* p.R337H carriers. Erythrocytes may be highly susceptible to oxidative stress because their membranes are rich in polyunsaturated fatty acids and because of their high oxygen and iron content [39]. In contrast, we did not detect significant differences in CAT and SOD activities between p.R337H carriers and non-carriers. Since our methodology for assessing SOD activity did not distinguish between different enzyme isoforms, further studies are needed to determine the extent of altered redox enzyme activities in the blood of *TP53* mutation carriers. Furthermore, we observed a significant decrease in ascorbic acid content among the carriers, which was associated with an overall increase in TAS. However the TAS assay was not informative of which anti-oxidant factor was responsible for this effect. One hypothesis to explain these findings is that an increase in endogenous antioxidants may arise to counterbalance the observed increased oxidative damage, while exogenous, diet-dependent antioxidants such as ascorbic acid are consumed.

It is noteworthy that the only homozygous p.R337H mutant subject who was included in this study was biochemically indistinguishable from the heterozygous mutation carriers. This finding was at first counter-intuitive since this patient did not have a wild type *TP53* allele that may have to some extent compensated for the loss of p53. However, our observation was compatible with the clinical history of this patient, who had a clinical phenotype equally undistinguishable from that observed in heterozygous mutation carriers. In fact, biochemical and structural studies on the p.R337H mutant protein are consistent with the hypothesis that in heterozygotes, the mutant p.R337H protein may have a dominant-negative effect over the wild-type allele, thus exerting essentially the same loss-of-function effects in either a heterozygous or homozygous context. Structural analysis of a peptide that encompasses the oligomerization domain of p53 revealed that the mutant retains the capacity to oligomerize and thus potentially behaves like wild-type p53 at pH 7.0 but looses this property at pH 8.0 due to the incapacity of histidine to donate an intermolecular hydrogen bond, which is required for p53 dimerization [22]. This pH effect is likely to have identical consequences irrespective of the wild-type or mutant nature of the protein that receives this hydrogen bond, thus providing a plausible explanation for the absence of a compounding effect in the homozygote p.R337H carrier.

In summary, this pilot study provides the first evidence that oxidative damage to lipids and proteins, and increased erythrocyte GPx activity, as well as increased total antioxidant status TAS, occur in carriers of a germline *TP53* mutation. The increased TAS suggests that oxidative stress in these subjects is partially compensated by the increased production of endogenous antioxidants. Whether this imbalance is specific to the *TP53* p.R337H mutation or is a general feature of *TP53* mutation carriers, whatever the mutation type, remains to be determined. Furthermore, it is not known whether the observed imbalance is associated with deregulated cell bioenergetics and/or with increased inflammatory stress, effects that may result from loss of wild-type p53 function. An abnormal oxidative profile was observed regardless of age and cancer diagnosis. If confirmed in a larger series of patients, and extended to a wider panel of markers of oxidative stress, these findings may have important implications on our understanding of the mechanisms of carcinogenesis associated to the *TP53* p.R337H mutation. Further studies involving interventions to correct or minimize this biochemical phenotype (i.e. supplementation with antioxidants) may identify novel strategies to reduce cancer risk in these subjects.

## Supporting Information

Table S1 Clinical characterization of the families *TP53* p.R337H mutation carriers and non-carriers.
(DOC)

Table S2 Comparison between p.R337H homozygous patient and to those identified in p.R337H/WT heterozygotes for the parameters evaluated.
(DOC)
