# Supplementary material for: Increased Oxidative Damage in Carriers of the Germline TP53 p.R337H Mutation
Source: PLoS One. 2012 Oct 9;7(10):e47010. doi: 10.1371/journal.pone.0047010 (PMC3467233; doi:10.1371/journal.pone.0047010)
Supplement: Table S1 — Clinical characterization of the families TP53 p.R337H mutation carriers and non-carriers. (DOC) [file pone.0047010.s001.doc]

**Table S1**

| **Genotype** | **Family** | **Gender** | **Pediatric (P) or Adult (A)** | **Cancer diagnoses** | **Age at diagnoses (in years)** |
| --- | --- | --- | --- | --- | --- |
| 1 – R337H/R337H | F1 | F | P | ACC | 1 |
| 2 – WT/R337H | F1 | F | A | - | - |
| 3 – WT/R337H | F2 | F | A | DBC | 35 |
| 4 – WT/R337H | F2 | F | A | - | - |
| 5 – WT/R337H | F2 | M | A | - | - |
| 6 – WT/WT | F2 | F | A | - | - |
| 7 – WT/R337H | F2 | F | P | ACC | 0.5 |
| 8 – WT/WT | F2 | F | P | - | - |
| 9 – WT/WT | F2 | F | A | - | - |
| 10 – WT/R337H | F2 | M | A | - | - |
| 11 – WT/R337H | F2 | M | P | - | - |
| 12 – WT/WT | F2 | M | P | - | - |
| 13 – WT/R337H | F2 | M | A | - | - |
| 14 – WT/R337H | F2 | F | A | - | - |
| 15 – WT/WT | F2 | M | A | - | - |
| 16 – WT/WT | F2 | M | A | - | - |
| 17 – WT/R337H | F3 | M | P | ACC | 1.10 |
| 18 – WT/R337H | F3 | M | P | - | - |
| 29 – WT/R337H | F3 | F | A | - | - |
| 20 – WT/R337H | F4 | M | P | CPT | 1 |
| 21 – WT/R337H | F4 | F | A | - | - |
| 22 – WT/WT | F4 | M | A | - | - |
| 23 – WT/WT | F4 | F | A | - | - |
| 24 – WT/R337H | F5 | F | A | - | - |
| 25 – WT/R337H | F5 | F | A | - | - |
| 26 – WT/WT | F5 | F | A | - | - |
| 27 – WT/WT | F5 | M | A | - | - |
| 28 – WT/WT | F6 | M | A | - | - |
| 29 – WT/WT | F6 | F | A | - | - |
| 30 – WT/WT | F6 | M | P | ACC | 0.10 |
| 31 – WT/WT | Not related | F | P | GCT | 13 |
| 32 – WT/WT | Not related | F | P | MT | 5 |
| 33 – WT/WT | Not related | M | P | ALL | 3 |
| 34 – WT/WT | Not retated | M | P | AML | 13 |

M=male and F=female

A=adult and P=pediatric

ACC (adrenocortical carcinoma), CPT (choroid plexus carcinoma), DBC (ductal breast carcinoma), ALL (acute lymphocytic leukemia), AML (acute myeloid leukemia), GCT (germ cell tumor) and MT (mature teratoma)
