# Supplementary material for: Increased Oxidative Damage in Carriers of the Germline TP53 p.R337H Mutation
Source: PLoS One. 2012 Oct 9;7(10):e47010. doi: 10.1371/journal.pone.0047010 (PMC3467233; doi:10.1371/journal.pone.0047010)
Supplement: Table S2 — Comparison between p.R337H homozygous patient and to those identified in p.R337H/WT heterozygotes for the parameters evaluated. (DOC) [file pone.0047010.s002.doc]

**Table S2**

|  | p.R337H Homozygous (n) | p.R337H ̸ WT Heterozygotes (n) | *P* |
| --- | --- | --- | --- |
| SOD - Erythrocytes | 12.25 (1) | 7.82 ± 0.85 (12) | 0.157 |
| CAT - Erythrocytes | 30.01 (1) | 23.75 ± 0.79 (15) | 0.059 |
| GPx - Erythrocytes | 7.70 (1) | 9.32 ± 0.86 (16) | 0.640 |
| SOD - Leukocytes | 39.00 (1) | 40.02 ± 0.63 (16) | 0.679 |
| GPx - Leukocytes | 12.59 (1) | 7.83 ± 0.78 (15) | 0.139 |
| SOD - Plasma | 31.25 (1) | 31.70 ± 0.53 (16) | 0.853 |
| SOD - Serum | 7.01 (1) | 7.38 ± 0.55 (10) | 0.825 |
| Ascorbic acid content - Plasma | 2.11 (1) | 2.35 ± 0.15 (15) | 0.700 |
| TAS - Plasma | 1.53 (1) | 1.47 ± 0.04 (15) | 0.695 |
| Carbonyl content - Plasma | 0.56 (1) | 0.45 ± 0.11 (10) | 0.772 |
| Carbonyl content - Serum | 1.25 (1) | 1.53 ± 0.27 (11) | 0.821 |
| MDA levels - Plasma | 161.3 (1) | 160.2 ± 0.97 (15) | 0.778 |

Data are presented as mean ± S.E.M. Enzyme activities are expressed as enzyme units (U)/mg protein. Ascorbic acid and malondialdehyde content in mM. Carbonyl content in nmol of carbonyl/mg protein and TAS in mmol/L.
